# Transimulation - Protein Biosynthesis Web Service

**DOI:** 10.1371/journal.pone.0073943

**Published:** 2013-09-05

**Authors:** Marlena Siwiak, Piotr Zielenkiewicz

**Affiliations:** 1 Department of Bioinformatics, Institute of Biochemistry and Biophysics, Polish Academy of Sciences, Warsaw, Poland; 2 Laboratory of Plant Molecular Biology, Faculty of Biology, Warsaw University, Warsaw, Poland; Weizmann Institute of Science, Israel

## Abstract

Although translation is the key step during gene expression, it remains poorly characterized at the level of individual genes. For this reason, we developed Transimulation – a web service measuring translational activity of genes in three model organisms: *Escherichia coli*, *Saccharomyces cerevisiae* and *Homo sapiens*. The calculations are based on our previous computational model of translation and experimental data sets. Transimulation quantifies mean translation initiation and elongation time (expressed in SI units), and the number of proteins produced per transcript. It also approximates the number of ribosomes that typically occupy a transcript during translation, and simulates their propagation. The simulation of ribosomes’ movement is interactive and allows modifying the coding sequence on the fly. It also enables uploading any coding sequence and simulating its translation in one of three model organisms. In such a case, ribosomes propagate according to mean codon elongation times of the host organism, which may prove useful for heterologous expression. Transimulation was used to examine evolutionary conservation of translational parameters of orthologous genes. Transimulation may be accessed at http://nexus.ibb.waw.pl/Transimulation (requires Java version 1.7 or higher). Its manual and source code, distributed under the GPL-2.0 license, is freely available at the website.

## Introduction

For many years, it was believed that gene expression regulation takes place mainly at the level of transcription. Nevertheless, upon the discovery that the mRNA transcription level can explain only 20–40% of the observed amounts of proteins [1], [2], the focus has been shifted to post-transcriptional mechanisms of gene expression regulation [3]–[5]. Although deeper insight into protein biosynthesis seems crucial to better integrate transcriptomic and proteomic data [6]–[8], the process is still poorly characterized at the level of individual proteins, mainly due to difficulties in experimental determination of absolute translation rates.

For this reason, we have developed [9] a model measuring translational activity at the level of individual genes, and implemented it genome-wide in *Saccharomyces cerevisiae*. Although the model is universal and can be used to study translation in any organism with a known genome, new implementations require careful selection of input data and numerous calculations. To address this issue, we decided to extend the set of results by applying the model to two additional organisms: *Escherichia coli* and *Homo sapiens* (HeLa cell line), for which high quality data sets on mRNA relative abundance, ribosome footprints, and tRNAs decoding specificities are available. Based on them, we calculated the absolute times of translation (elongation and initiation separately), in SI units, for individual genes. Furthermore, by combining these results with data on mRNA stabilities, we determined the number of proteins produced from each transcript during its lifetime.

To facilitate access to the results, we developed Transimulation – a web service simulating protein biosynthesis from individual genes for the three studied organisms. Transimulation not only provides a graphical interface for browsing and searching for gene products and displays the outcome in a transparent fashion, but also simulates the average propagation of ribosomes on an mRNA molecule according to the calculated translational parameters of a gene. The visualization of ribosome density on a transcript enables detection of regions most susceptible to ribosome collisions and queuing. Moreover, the movement of ribosomes may be modified on the fly by coding sequence manipulation. The users may introduce any number of point mutations into the coding sequence, both synonymous and non-synonymous, in order to examine their impact on the fluency of ribosome flow. Transimulation also enables uploading of any coding sequence and expressing it *in silico* in one of the three analyzed organisms. In such a case, the expected time of translation initiation cannot be determined on the basis of experimental data and must be provided by the user. This functionality of the web service may be of crucial importance for studies on heterologous expression. Finally, Transimulation enables large-scale analysis of genes as translational parameters for all analyzed genes, their coding sequences, and mean codon elongation times for an organism can be easily downloaded as flat files. We demonstrated how Transimulation may be used to examine evolutionary conservation of translational parameters in orthologous genes.

## Results

### Translational Model for Three Organisms

The following translational parameters were attributed to the analyzed genes: 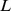, coding sequence length in codons; 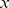, average number of transcripts in a cell; 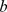, average number of proteins produced from one molecule of transcript during its lifespan; 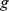, ribosome density in number of ribosomes attached to a transcript per 100 codons; 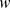, the absolute number of ribosomes on a transcript; 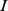, mean time required for translation initiation; 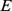, mean time required for translation elongation; 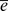, mean elongation time of one codon of a transcript; and 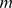, estimated mean lifetime of a transcript. Parameters 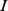, 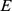, 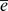, and 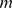 are given in SI units.

The values of parameters 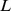, 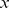, 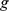, 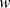, 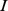, 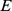, and 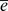 were attributed to 1738, 4470, and 7494 genes in *E.coli*, *S.cerevisiae*, and *H.sapiens*, respectively, which corresponds to the 42, 76, and 41% coverage of the genomes. Due to the accessibility of data, parameters 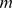 and 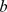 were determined only for subsets of analyzed genes, containing, respectively, 1574, 3425, and 6205 genes. The summary of quantitative measures of translation for the three organisms may be found in Table 1.

**Table 1:** The summary of translational parameters calculated in the model.

organism		*L*	*x*	*b*	*g*	*w*	*I*	*E*	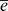	*m*
*E.coli*	mean	335	3.6	47	1.3	4.0	62	40	119	7.5
	median	298	1.7	28	0.8	2.3	15	35	119	6.8
	sd	203	5.6	60	1.3	5.0	206	24	9	4.0
	min	15	0.1	0	0	0	2	2	87	2.0
	max	1487	54.0	940	6.6	41.2	5091	178	177	42.3
*S.cerevisiae*	mean	513	7.8	116	1.1	5.6	54	116	224	33.2
	median	431	2.7	58	0.8	3.1	28	96	229	27.4
	sd	365	29.0	188	0.9	7.3	186	84	31	26.8
	min	37	0.1	1	0.0	0.0	2	4	98	4.3
	max	4911	591.3	2543	6.6	142.1	6714	1074	360	677
*H.sapiens*	mean	676	85.9	9171	2.3	11.5	7	59	87	6.5
	median	506	42.6	5616	2.1	10.1	4	44	87	9.2
	sd	620	171.9	9739	1.4	7.22	23	54	4	6.3
	min	38	0.9	14	0.0	0.0	1	3	75	3.0
	max	14508	4e3	83e3	7.5	131.6	1372	1232	108	34.6

Column description: (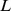) transcript length; (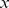) number of gene transcripts; (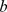) number of proteins produced from one transcript; (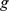) ribosome density in number of ribosomes per 100 codons; (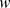) number of ribosomes on a transcript; (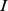) initiation time in s; (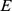) elongation time in s; (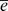) mean elongation time of one transcript codon in ms; and (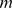) mean transcript lifetime in min (bacteria, yeast), or in h (humans). For all parameters, except 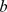 and 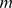, the rows 1–15 were calculated for 1738, 4470, and 7494 genes for bacteria, yeast, and humans, respectively. For parameter 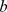 and 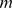, the rows were calculated for 1574, 3425, and 6205 genes, respectively.

### Web Service Implementation

All translational parameters for individual genes are presented on the Transimulation website. The database may be browsed or searched by the query engine. Simple searches may be performed by typing a single gene name, or a key word in the query window. More complicated queries, combining several gene names, key words, or values and ranges of translational parameters are also possible. The results page of an individual gene consists of a list of all the calculated translational parameters and an interactive simulation of translation.

**Figure 1:**
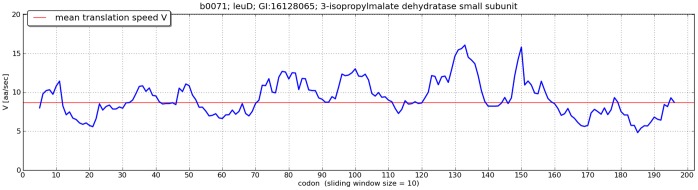
Translation speed plot generated by Transimulation. An example plot of translation speed (in aa/sec) in relation to the coding sequence of one of the *E.coli* genes. To facilitate analysis, the plot was smoothed by calculating translation speed over a 10-codon sliding window. Similar plots for window sizes of 1, 2, 5, 10, 20, 30, and 50 codons are generated for all analyzed genes and sequences uploaded by the user.

The top part of the applet displays transcript coding sequence, which may be navigated or mutated with the help of appropriate buttons on the control panel. The current sequence may be downloaded as a fasta file anytime. The simulation starts by placing the ribosome active site on the initial codon and then moving it from one codon to another only if it has spent there the required amount of time for translation of the current codon and the subsequent codon is vacant. The successive ribosome attaches to the initial codon after a time interval equal to the translation initiation time. Ribosome collisions will not occur during simulation, if the original sequences are used, as only collision-free genes were retained in the database. Otherwise, ribosome blockage may take place. In such a case, the simulation stops and active sites of collided ribosomes are indicated by red exclamation marks. For easier identification of ribosome deceleration regions, plots of translation speed (in aa/sec) in relation to the original coding sequence are provided. To facilitate analysis, the plots may be smoothed by calculating translation speed over a sliding window of the size of 2, 5, 10, 20, 30 or 50 codons (see Figure 1).

In addition, Transimulation allows to express any coding sequence *in silico* in one of the three studied organisms. To run simulation of ribosome movement, the time of translation initiation should also be provided by the user. Translation times of individual codons are those of the host organism. Most translational parameters are calculated based on the initiation time and the coding sequence. To calculate the number of proteins produced per mRNA – 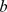, mean lifetime of a transcript should also be provided. Additionally, translation speed plots (raw and smoothed) may be generated for the uploaded sequence. Finally, to facilitate more automatic analysis, the entire database of translational parameters, as well as translation times of individual codons, may be downloaded as flat files. Detailed manual for Transimulation users may be found on the website.

### Agreement with Previous Studies

A detailed comparison of model results with other studies was shown previously for yeast [9]. However, as in this study we used different input data set for yeast mRNA degradation time [10], which is a key parameter for calculating 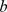, we repeated the comparisons of obtained protein abundances with experimental data. Similar but more detailed analysis for bacteria and humans was also performed.

**Figure 2:**
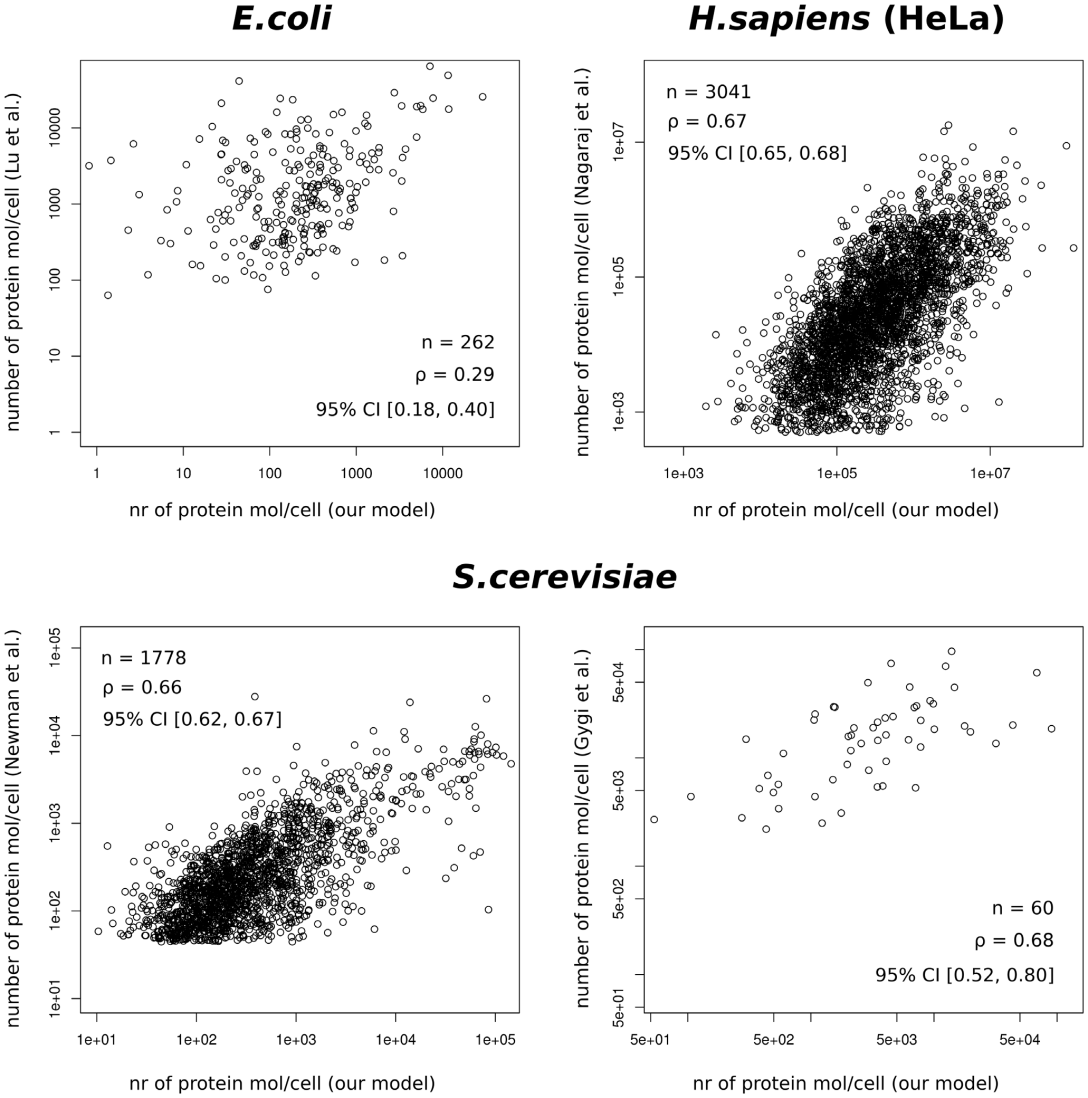
Calculated protein abundance vs experimental studies. Correlations between protein abundances calculated in our model (as 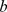 times 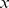) and those obtained in experimental studies [8], [11]–[13]; n – sample size, 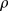 – Spearman correlation coefficient and its 95% confidence interval.

At first, we examined the compatibility of our predictions with genome-wide, experimental measurements of protein levels for *E.coli* [11], *S.cerevisiae* [8], [12], and *H.sapiens* [13]. All scatter plots and distributions of log fold differences are presented in Figure 2 and S1, respectively. For *H.sapiens* the 95% confidence interval (CI) for the Spearman correlation coefficient 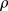 is 0.65–0.68 (for sample size n = 3041), which means that our model explains 42–46% of the variability of protein levels measured experimentally. Globally, our predictions are overestimated by approximately one order of magnitude in relation to experimental studies. Next, we compared protein abundances in *S.cerevisiae* with those assessed experimentally by other groups [8], [12]. The obtained 95% CI for 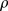 was 0.62–0.67 (n = 1778), and 0.52–0.80 (n = 60) for the Newman et al. and Gygi et al. data sets, respectively. Our predictions explain 38–45% and 27–64% of the experimental values’ variability in these data sets, and may be slightly shifted in relation to them, although the difference rarely exceeds one order of magnitude. For comparison, the 95% CI for 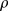 calculated between these two experimental studies is 0.35–0.64 (n = 97), which corresponds to 12–41% of each other’s explained variability. For *E.coli*, the 95% CI for 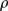 was 0.18–0.40 (n = 262), indicating that our model explains 3–16% of the variability of protein abundances measured experimentally. Again, there is a shift in values, but this time our protein levels are underestimated; for some genes this shift may be serious (several orders of magnitude), as may be seen from the long left tail of the log fold differences distribution (Figure S1). For explanation of this fact, see Discussion.

Furthermore, many of the obtained cell-wide parameters of translation can be compared with other quantitative studies. For instance, according to our model the global ribosome density calculated over the entire *E.coli* transcriptome equals 3.46 ribosomes per 100 codons. Assuming that a ribosome covers 10 codons, the length of average gap between ribosomes equals ∼19 codons, which corresponds to an earlier report [14], claiming 14–28 codons between adjacent ribosomes. Additionally, the average translation rate for bacteriae cells was estimated at 12–21 [14], and 15 aa/sec [15], while our model predicts 8.4 aa/sec. Our mean transcript lifetime of 7.5 min for *E.coli* agrees well with previous estimates of 5.87 min ± 34 s [16] and 5–10 min [17]. Besides, Open WetWare web page (http://openwetware.org, accessed Jan 2013) provides a list of *E.coli* statistics, which generally confirm our calculations of: transcript copy number per gene (2–3 according to Open WetWare vs. 1.7 in our model [median]); translation initiation time (20–30 vs. 15 s in our model [median]); the number of proteins produced from one transcript (40 vs. 28 [median] and 48 [mean] in our model). There is a discrepancy in the mean number of proteins produced from a gene (1000 vs. 45 [median] and 205 [mean]). Other studies also claim that the total number of proteins in a bacterial cell is ∼2.4 mln [18], while our calculation (sum of 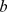 times 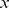 over all analyzed genes) gives only ∼300,000. The origins of all the discrepancies are analyzed in Discussion.

In HeLa cells, the global translational rate estimated by the model is ∼11 aa/sec. Previous studies reported 6 aa/sec for human apolipoprotein B [19], 0.74 aa/sec for rabbit hemoglobin [20], 5 aa/sec for chick ovalbumin [21], and an average translation rate of 7.3 aa/sec in cockerel liver [22]. Moreover, according to reference [23], the entire proteome of a mammalian cell contains about 8e9 molecules, while our model predicts 6e9 proteins per cell. The distribution of protein abundances is also in accordance with this report, claiming variation in protein levels from less than 100, to 1e8 molecules, depending on their function. In our model, the number of proteins produced from a gene (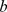 times 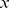) is between 127 and 1e8 molecules per cell, with median 230,000 and standard deviation 3.3e6.

### Case study: Comparison of Translation in Three Organisms

To demonstrate the applicability of Transimulation to answering biological questions we used its data to estimate conservation of translational parameters between evolutionary related genes. By taking advantage of the Inparanoid database [24] (accessed Jan 2013), we prepared a list of 69 orthologous genes present in *E.coli*, *S.cerevisiae* and *H.sapiens* genomes, for which translational parameters can be found on the Transimulation website (Table S1). We measured the agreement between parameters values for all possible pairwise comparisons, by calculating 95% confidence intervals CI for the Spearman correlation coefficient 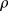. All results, as well as three-dimensional scatter plots, are provided in Figure S2.

The closest agreement, yet still relatively small, was found for the transcript copy number 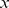. For intra-species comparisons, all correlations were positive, with lower CIs limits between 0.13–0.29, and upper CI limits between 0.55–0.65, indicating that the percent of explained variability of mRNA levels is in the range 2–42. Although this is not much, and could result from data noise, we must not forget that the analyzed species are very distant, and stronger signals are hardly expected. No coherent picture emerges from comparisons of parameters 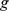, 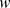, 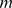, 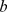, and 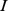, as obtained confidence intervals are too ambiguous to decide on correlation sign, thus precluding any further discussion. The only exception is the case of the yeast-human comparison for parameters 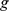 and 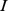, for which some positive correlations were found. Here again, the signal is weak, possibly explaining as little as 4–5% of the values variability. The number of ribosomes attached to a transcript 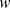 correlates better, with lower CI limit of 0.43, corresponding to at least 18% of the explained variability. However, this is the result of strong conservation of orthologs’ sequence length 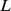, which significantly affects the calculations of 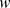. This influence is also visible in elevated CIs for the remaining intra-species comparisons of 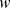.

Translation elongation time 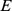 seems strongly conserved, with correlation CI lower limits ranging from 0.7 to 0.83, indicating that at least 49% of values variability could be explained. Nevertheless, this is again due to conservation of 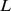, the main determinant of total elongation time. In particular, the inter-species comparisons for the samples of 69 genes show very strong correlations between 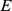 and 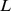, with 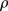 above 0.83 (yeast), 0.96 (bacteria), and even 0.99 (humans). Furthermore, this is confirmed by unambiguous correlations of 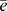, mean elongation time of one codon of a transcript, suggesting that in case of evolutionary related genes of similar sequence (and thus sequence length) any variability of elongation times of individual codons (or codons substitutions, insertions, and deletions) has negligible effect on total elongation time of the transcript. The differences in elongation times of individual codons seem too small to significantly affect 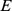 for sequences of similar length. Hence, it should not be surprising that the intra-species comparison of mean elongation times of 61 sense codons (all measured at 37°C) results in very weak (if any) positive correlations. However, this may also be explained by the independent adaptation of species to the changes in tRNAs pool in the cell.

Taken together, our results suggest rather modest conservation of transcript copy number in orthologs. We cannot exclude the possibility that ribosome density and initiation time is also slightly conserved, although it could not be confirmed by comparisons with *E.coli*. Possibly, the evolutionary distance between bacteria and analyzed eukaryotes is too large to detect such a weak signal. We could not detect any conservation of mean transcript lifetimes and the number of produced proteins per transcript, which may stem from the fact that in the course of evolution many of the analyzed genes duplicated, gained or lost function. In consequence, their stoichiometry in the cell may be very distant from that in the theoretical common ancestor of these three species. Finally, the observed conservation of total elongation time 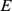 of orthologs should rather be due to similar sequence length of analyzed genes.

## Discussion

The Transimulation service provides easy access to the results of the computational model of translation applied cell-wide in three organisms: *E.coli*, *S.cerevisiae*, and *H.sapiens*, and also enables to simulate translation of individual genes, including arbitrary sequences provided by the user. It is freely available at http://nexus.ibb.waw.pl/Transimulation. The simulation of ribosomes’ movement is written in the Java programming language and requires Java version 1.7 or higher plugged into the browser. The source code is distributed under the GPL-2.0 license and may be freely downloaded from the Transimulation website, along with installation instructions and all the necessary input data.

The results presented in this and our previous paper [9] show that generally the predictions of our model are reasonably good, taking into account the differences in strains and experimental conditions as well as assumptions and simplifications of the model. However, some discrepancies can be found, especially in protein abundance. In case of humans, they may be assigned to the fact that our model does not take into consideration protein turnover, and, therefore, the experimentally observed protein level should be smaller than predicted, especially for short-lived proteins. In contrast, in *E.coli* our predictions are strongly underestimated, which most likely stems from the fact that *E.coli* has a very short generation time, varying between ∼18 and ∼38 min for rich and minimal medium, respectively [25]. Simultaneously, the vast majority of its proteins has much longer half-life. For instance, it was reported that only 2 to 7% of the proteome degrades at half-life as short as ∼1 hour [26]. This means that a typical protein lifetime strongly exceeds cell generation time, and protein molecules are inherited by subsequent generations through cell devisions. Summing up, we do not expect very good agreement between 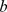 – the average number of proteins produced from one molecule of transcript during its lifespan – and experimentally determined protein concentrations, as protein concentration in a cell is not exactly what 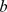 stands for.

Nevertheless, this problem shows difficulty of evaluation of the model at the current level of biological knowledge. For individual genes, most parameters, such as translation time and protein production rate, cannot be compared with experimental studies, because no such studies exist or are available only for a very limited set of genes. Even genome-wide determinations of mRNA or protein levels, performed by several groups separately (e.g. in yeast), are far from setting the gold standard [27]. In particular, our predictions for yeast proteins abundance show similar agreement with experimental studies, as the experimental studies among themselves. However, one must not forget that those experimental studies can explain even as little as 12% of their counterpart variance. Some researchers argue [27] that many genome expression data sets suffer from large random errors and systematic shifts in reported values, and thus cannot be used to predict translation rates at the level of individual proteins. Nevertheless, even if the experimental procedures were more precise, we would not avoid the variability of the measurements due to the fact that the cell is alive and thus constantly interacts with the environment. Hence, only numerous repetitions of quantitative genome-wide experiments in fixed conditions, but performed separately, could provide enough data for complex and comprehensive meta-analysis of gene expression and a good estimate of the errors. Only such data could provide stable ground for translational models, like the one presented above, finally upgrading all estimations from point to interval-oriented. For this reason, we recommend taking any parameter value at the level of individual gene with a much caution. We hope to develop and complete Transimulation as more genome-wide data become available, so that it becomes a theoretical framework for a future more predictive quantitative model.

## Materials and Methods

### Computational Model of Translation

The model used to calculate the translational parameters presented in our web service has been described in detail previously [9]. The majority of the input data and other variables required for the model were found in the Bionumbers database [28], and are presented in Table 2. All translational parameters for yeast, except 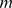 and 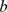, were taken directly from Table S1 of [9]. Below, we briefly summarize the calculations, along with some details on data sets parsing for the remaining organisms. More thorough derivation of the equations may be found in our previous work [9].

**Table 2:** Summary of data sets and variables used as an input of the model.

Input data	*E.coli*	*S.cerevisiae*	*H.sapiens*
Cell line	K12 MG1655	BY4741	HeLa
Temperature	37°C	30°C	37°C
Medium	MOPS	YEPD	–
**Global parameters**			
Transcriptome size	1,500 [15], [35]	36,000 [36]	700,000*
Ribosomes/cell	20,000 [15]	200,000 [37]	9,500,000 [38]
Average cell volume	1e-18 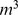 [29]	42e-18 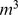 [39]	2425e-18 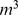 [40]
**Parameters required to calculate mean codon elongation times**			
tRNA decoding	[29]	[41]	[42]
tRNA abundances	[43]	[9]	[42]
tRNAs/cell	71,000 [43]	2,800,000 [9]	60,000,000*
**Data sets**			
Coding sequences	NCBI	SGD	UCSC
mRNA abundances	[11]	[44]	[45]
mRNA lifetime	[46] (M9 medium)	[10]	[47]
Ribosome footprints	[34]	[44]	[45]

Details on data parsing and calculations may be found in the main text. Cell lines and growth conditions (temperature and medium) denote those used in the ribosome profiling experiments. The numbers marked by an asterix were taken from the RNA Tools and Calculators section at the Invitrogen Website (www.invitrogen.com, accessed April 2013). The coding sequences were downloaded from the following databases: NCBI (www.ncbi.nlm.nih.gov.ftp, accessed May 2012), SGD (www.yeastgenome.org, accessed June 2009), and UCSC (http://genome.ucsc.edu, accessed July 2012).

### Coding Sequences

Coding sequences of the analyzed organisms were downloaded from the web resources shown in Table 2. Our reference genomes were the same as those used in the ribosome profiling analysis of the species, i.e. NC_000913 for *E.coli*, and hg18 for humans.

### Codon Elongation Times

Mean elongation times of individual codons of *E.coli* were taken directly from [29], and for yeast from [9]. For humans, we obtained them as described in [29]. In short, the average time to add an amino acid coded by the 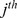 codon to the nascent peptide chain was calculated as stated in [29], namely:
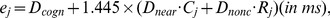
(1)where 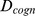 is the average time to insert an amino acid from a cognate aa-tRNA, and 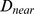 and 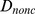 are the average time delays caused by the binding attempts of near- and non-cognate aa-tRNAs, respectively. Values of 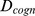, 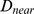 and 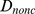 can be calculated at any given temperature, as shown by [29]. In our analysis we used the same temperatures at which the cells were grown in the ribosome profiling experiments: 30°C for yeast, and 37°C for *E.coli* and humans. In the above equation 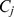 and 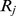 stands for two tRNA competition measures, being the quotients of the sums of arrival frequencies of near-cognates vs. cognates and non-cognates vs. cognates, respectively. For each codon we determined its cognates, near- and non-cognates, based on data sets on tRNA specificities listed in Table 2. We assume that all sense codons have one or more cognate aa-tRNA and varying numbers of near-cognates. Near-cognates are defined as having a single mismatch in the codon-anticodon loop in either the 2nd or 3rd position. Since some cognate tRNAs have a mismatch in the 3rd position, these tRNAs are excluded from the set of near-cognates [30]. The arrival frequency of the aa-tRNA molecule is defined as in [29]:
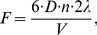
(2)where 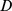 is the diffusion coefficient, 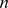 is the number of molecules in a cell, 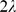 is the molecule size in m, and 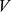 is the average cell volume in m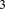. The values of 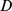 for all aa-tRNAs were taken directly from [29]. As this value depends only on the accepted amino acid, we assumed that the difference in size between E.coli and other species’ tRNAs is negligible. For humans the diffusion coefficient of the tRNA(Cys) was used for the selenocysteine isoacceptor tRNA(Sec). The levels of tRNA molecules in a cell, as well as their decoding specificities, were taken from sources given in Table 2. If necessary, the relative abundances were transformed to absolute values assuming the total number of tRNAs listed in Table 2. The values of 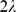 used previously [29] were determined separately for individual E. coli aa-tRNA molecules [31]. As we are not aware of any similar reports for other analyzed organisms, we decided to use 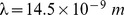 for other species’ codons, which is the mean of the E. coli 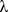 values. Average cell volumes for E.coli, yeast and humans were taken directly from the references in Table 2. Mean codon elongation times for all three species may be downloaded as flat files from the Transimulation web page, and parameters 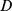, 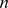 and 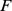 are presented in Table S2. The list of cognate and near-cognate tRNA for each codon, as well as the measures 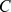 and 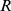, may be found in Table S3.

### Transcript Abundance

The levels of mRNA molecules were taken directly from the references in Table 2. If necessary, the relative abundances were transformed to absolute values assuming the total number of mRNAs given in Table 2 and complete coverage of the transcriptome by the reference study.

### Ribosome Density

The average number of ribosomes attached to a transcript – 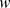, as well as the ribosome density 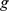 (the number of attached ribosomes per 100 codons), were determined on the basis of genome-wide ribosome profiling data, as stated in Table 2. For human and yeast, genes that did not have either ribosome or mRNA footprint counts at all, or their sum was below 128, were excluded from further analysis. As the *E.coli* data set does not provide information on mRNA counts, only genes with less than 100 ribosome footprint counts in at least one repetition were excluded. When transforming footprint counts into ribosome density for *E.coli*, one obstacle cannot be ignored – the fact that typically *E.coli* expresses ∼600 genes at a time from the pool of ∼4000 [15] and transcript turnover is very rapid [16], [17]. The ribosome profiling data provide information for ∼3000 genes, which means that it concerns bacterial cells at many possible stages. The key parameter for ribosome density calculation is the sum of all footprints from the experiment, which is assumed to correspond to the total number of footprints in a cell. In bacteria the sum calculated over all ∼3000 analyzed genes would be seriously overestimated. To overcome this problem, we estimated it by finding the mean of 1000 sums calculated over 600 genes, sampled without replacement from the pool of 3331 genes of known footprints count. For all species the total number of ribosomes required for calculations of 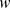 was taken from the references in Table 2, and it was assumed that 85% of ribosomes present in the cell actively participate in translation [32], [33]. Assuming that a ribosome covers about 10 codons [34], only transcripts with 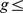 10 were retained.

### Other Translational Parameters

The average elongation time of a transcript 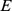 was calculated as the sum of mean elongation times of its codons. Transcript mean elongation time of one codon 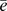 was calculated as 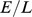, where 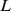 stands for sequence length in codons. The translation initiation time 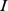 was calculated as the quotient of 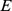 and the number of ribosomes attached to a transcript – 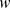, as discussed previously. Mean mRNA lifetimes were taken from the references listed in Table 2. The expected number of proteins produced from a transcript during its lifespan was calculated as the quotient of the mean lifetime and translation initiation time 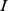. Using the simulation of ribosome movement on a transcript and calculated parameters, we reduced the final data set to the transcripts on which ribosome queuing does not occur. We excluded 89, 151 and 194 transcripts from *E.coli*, yeast and human data sets, respectively.

## Supporting Information

Figure S1 Distributions of log fold differences for comparisons of protein abundances calculated in our model and obtained in experimental studies.
(PDF)

Figure S2 Correlations of translational parameters values of orthologous genes common for three analyzed species.
(PDF)

Table S1 The list of 69 orthologous genes used in the analysis of evolutionary conservation of translational parameters.
(PDF)

Table S2 Decoding specificities of yeast and human tRNAs and calculated values of the model parameters.
(PDF)

Table S3The list of codons, their corresponding cognate and near-cognate tRNAs, and competition measures 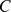 and 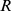.(PDF)
