# Supplementary material for: Transimulation - Protein Biosynthesis Web Service
Source: PLoS One. 2013 Sep 5;8(9):e73943. doi: 10.1371/journal.pone.0073943 (PMC3764131; doi:10.1371/journal.pone.0073943)

Figure S1: Distributions of log fold differences for comparisons of protein abundances calculated in our model (as b times x) and obtained in experimental studies.

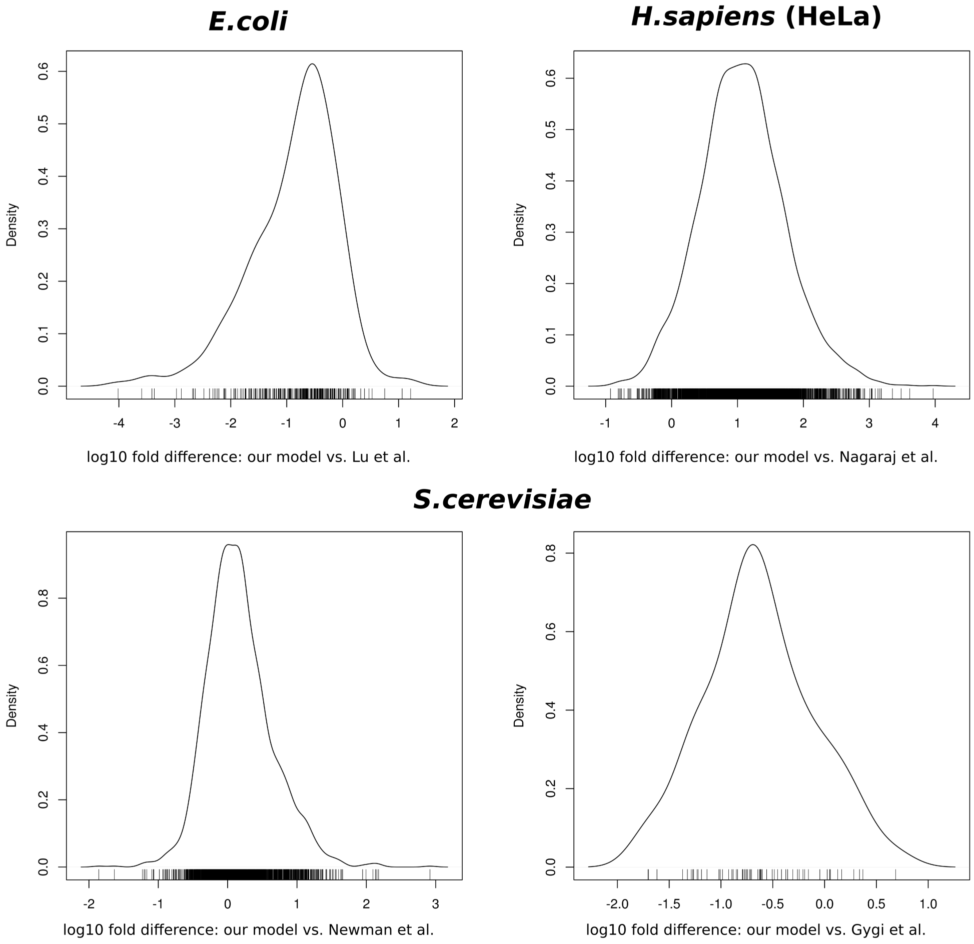

Supplement: Figure S1 — Distributions of log fold differences for comparisons of protein abundances calculated in our model and obtained in experimental studies. (PDF) [file pone.0073943.s001.pdf]
