# Supplementary material for: Transimulation - Protein Biosynthesis Web Service
Source: PLoS One. 2013 Sep 5;8(9):e73943. doi: 10.1371/journal.pone.0073943 (PMC3764131; doi:10.1371/journal.pone.0073943)

Figure S2: Correlations of translational parameters values of orthologous genes common for three analyzed species (plots 1-7 and 9-10), and correlations of mean elongation times of 61 sense codons calculated for three analyzed species (plot 8). All pairwise comparisons between species are presented. Left panels: 95% confidence intervals for Spearman correlation coefficients are plotted as horizontal lines, n stands for sample size. Right panels: three-dimensional scatter plots of compared parameters' values; if necessary, the values are logged.

1) **L - coding sequence length**

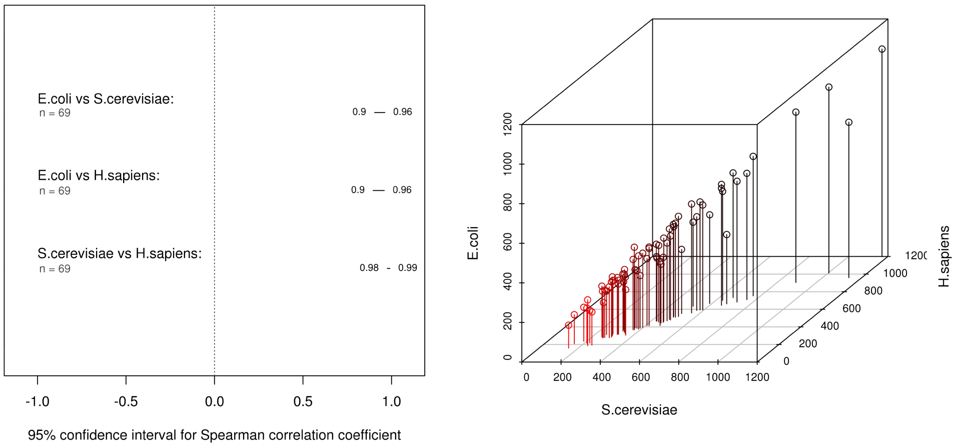

2) **x - transcript copy number**

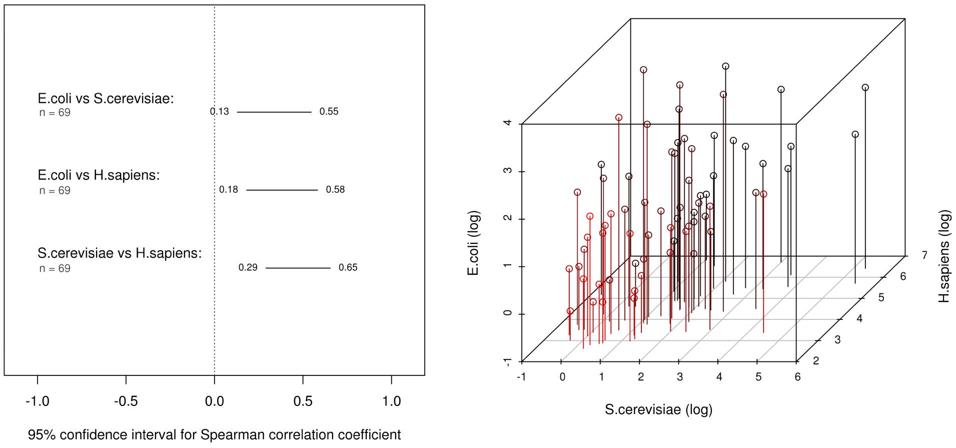

3) g - ribosome density

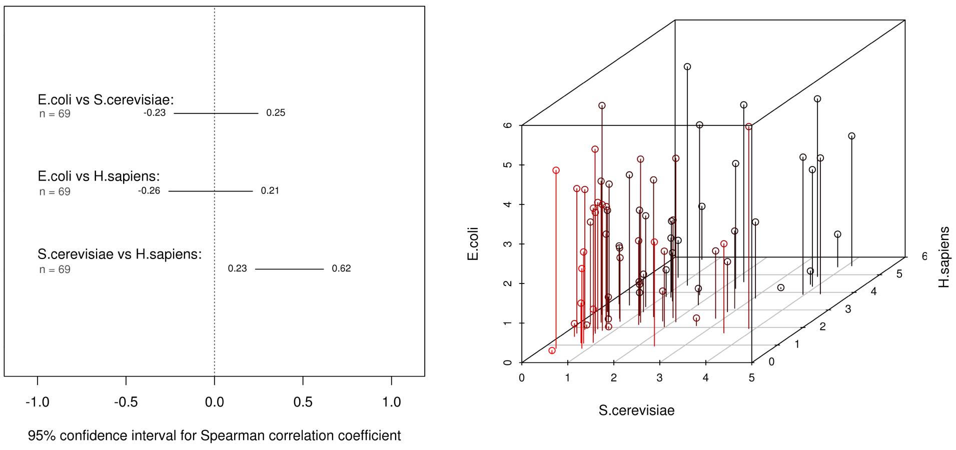

4) w - number of ribosomes per transcript

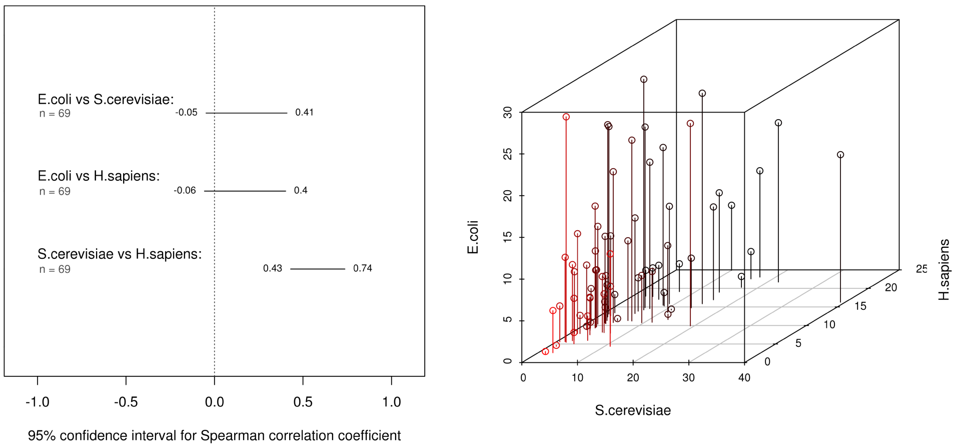

5) I - mean initiation time

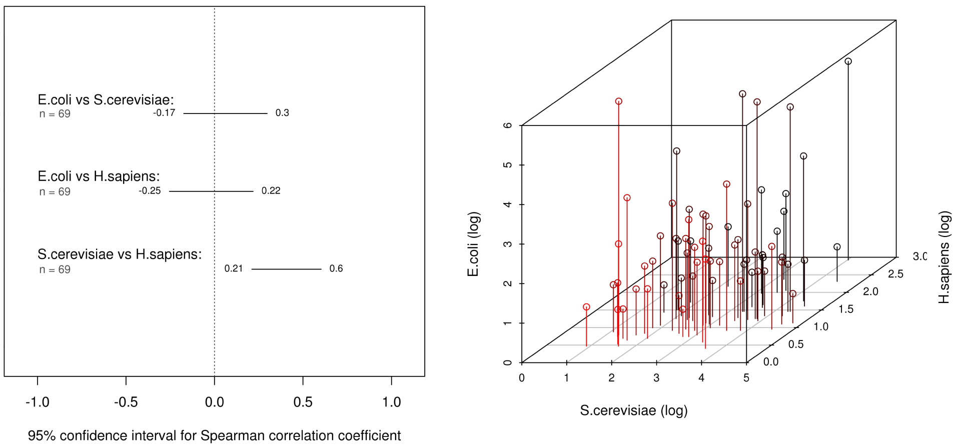

6) E - total elongation time

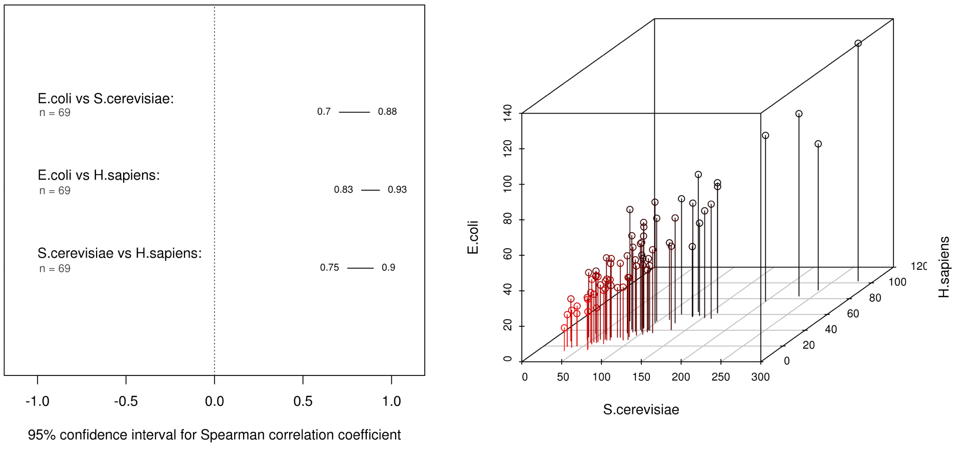

7) $\overline{e}$ - transcript mean elongation time of one codon

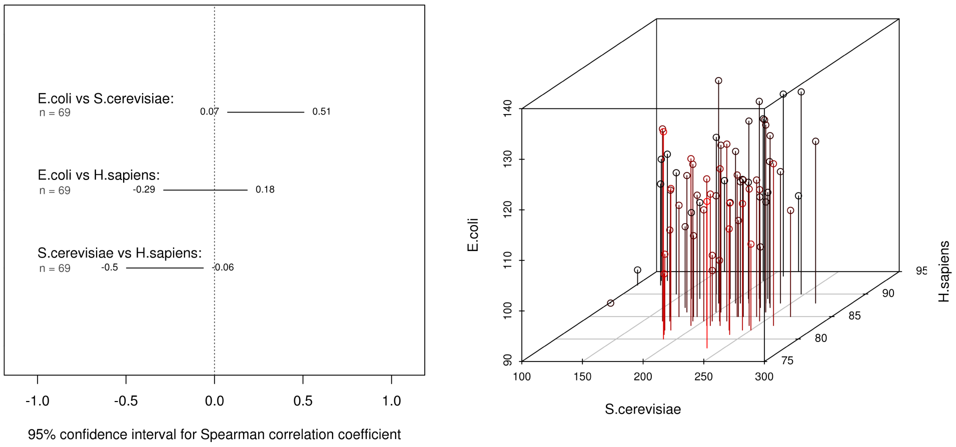

8) mean elongation times of 61 sense codons

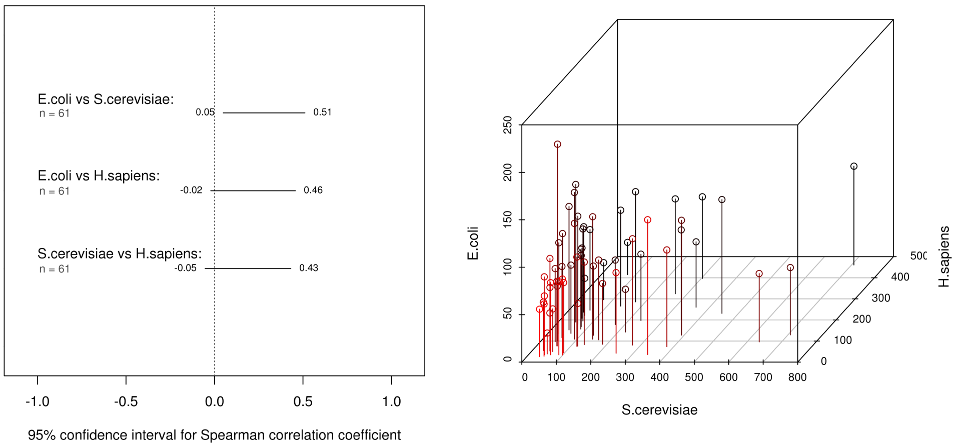

9)

m - transcript mean lifetime

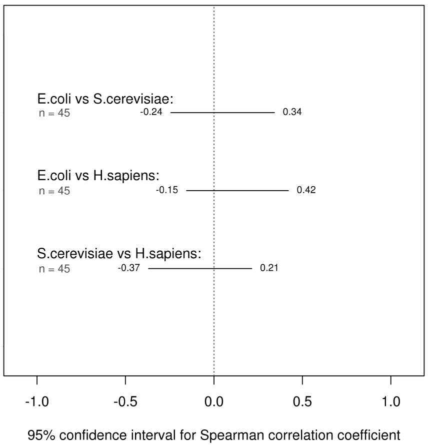

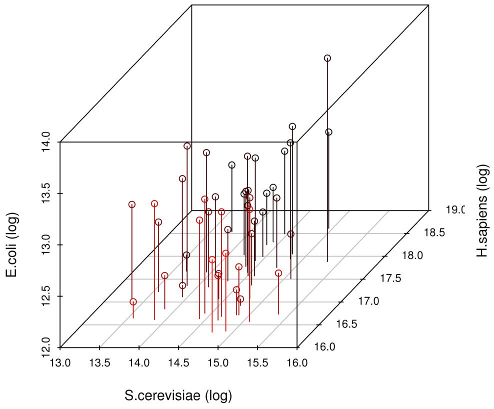

10)

b - number of proteins produced from one transcript

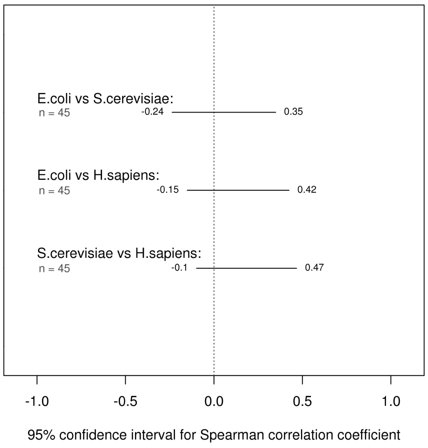

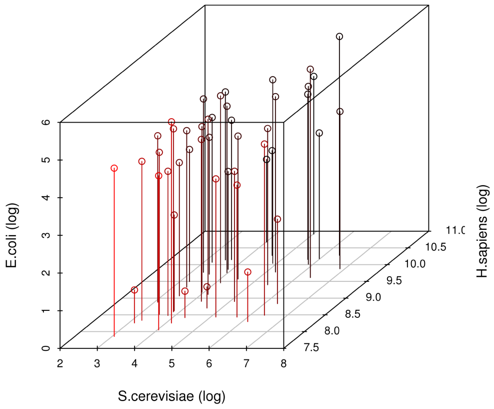

Supplement: Figure S2 — Correlations of translational parameters values of orthologous genes common for three analyzed species. (PDF) [file pone.0073943.s002.pdf]
