# Supplementary material for: Transimulation - Protein Biosynthesis Web Service
Source: PLoS One. 2013 Sep 5;8(9):e73943. doi: 10.1371/journal.pone.0073943 (PMC3764131; doi:10.1371/journal.pone.0073943)
Supplement: Table S1 — The list of 69 orthologous genes used in the analysis of evolutionary conservation of translational parameters. (PDF) [file pone.0073943.s003.pdf]

Table S1: The list of 69 orthologous genes used in the analysis of evolutionary conservation of translational parameters. The list was prepared on the basis of Inparanoid database (Ostlund et al. 2009).

S.cerevisiae	*H.sapiens*	*E.coli*
YBL022C	NM_004793	b0439
YBL036C	NM_007198	b2951
YBL039C	NM_001905	b2780
YBR003W	NM_014317	b3187
YBR196C	NM_000175	b4025
YBR227C	NM_006660	b0438
YCR012W	NM_000291	b2926
YDL055C	NM_013334	b2039
YDL104C	NM_022353	b3064
YDL168W	NM_000671	b0356
YDL219W	NM_080820	b3887
YDL236W	NM_001042371	b0675
YDR047W	NM_000374	b3997
YDR050C	NM_001159287	b3919
YDR298C	NM_001697	b3735
YDR304C	NM_000942	b0525
YDR456W	NM_001042537	b4065
YEL029C	NM_003681	b1636
YER023W	NM_006907	b0386
YER163C	NM_001008708	b1218
YFL018C	NM_000108	b0116
YFR009W	NM_018358	b3352
YFR031C	NM_001042550	b0397
YFR047C	NM_014298	b0109
YGL018C	NM_172002	b2527
YGL236C	NM_001123226	b3741
YGR155W	NM_000071	b2414
YGR244C	NM_003850	b0728
YGR255C	NM_182476	b2907
YJL026W	NM_001034	b2235
YJL035C	NM_182503	b2559
YJL060W	NM_001008661	b0600
YJL068C	NM_001984	b2154
YJR019C	NM_005469	b0452
YKL104C	NM_005110	b3729
YKL184W	NM_002539	b2838
YKR080W	NM_006636	b0529
YLL041C	NM_003000	b0724
YLR027C	NM_002079	b0928
YLR069C	NM_024996	b3340
YLR244C	NM_015143	b0168
YLR289W	NM_021927	b2569
YLR351C	NM_020202	b0219
YLR354C	NM_006755	b2464
YLR359W	NM_000026	b1131
YLR382C	NM_015340	b0642
YML021C	NM_003362	b2580
YML080W	NM_022156	b3260
YMR120C	NM_004044	b4006
YMR300C	NM_002703	b2312
YNL218W	NM_020135	b0892
YNR041C	NM_015697	b4040
YOL096C	NM_017421	b2232
YOR095C	NM_144563	b2914
YOR136W	NM_005530	b1136
YOR142W	NM_003849	b0729
YOR157C	NM_002799	b3932
YOR243C	NM_019042	b2745
YOR274W	NM_017646	b4171
YOR386W	NM_004075	b0708
YPL040C	NM_018060	b0026
YPL059W	NM_016417	b1654
YPL091W	NM_000637	b3500
YPL097W	NM_001040436	b1637
YPL104W	NM_018122	b1866
YPL172C	NM_001303	b0428
YPL262W	NM_000143	b1611
YPL266W	NM_014473	b0051
YPR047W	NM_006567	b1714
