# Supplementary material for: Transimulation - Protein Biosynthesis Web Service
Source: PLoS One. 2013 Sep 5;8(9):e73943. doi: 10.1371/journal.pone.0073943 (PMC3764131; doi:10.1371/journal.pone.0073943)
Supplement: Table S2 — Decoding specificities of yeast and human tRNAs and calculated values of the model parameters. (PDF) [file pone.0073943.s004.pdf]

Table S2: Decoding specificities of yeast and human tRNAs and calculated values of the model parameters. Column descriptions: (1) tRNA number; (2) codons recognized by a tRNA molecule; (3) tRNA anticodon; (4) coded amino acid; (5) D – diffusion coefficient of a aa-tRNA molecule in 10^{-11} m^2/s; (6) n – the number of tRNA molecules in a cell; and (7) F – arrival frequency of a aa-tRNA molecule to the vacant A site of a ribosome in s^{-1}. Similar table for *E.coli* may be found in the original work of Fluitt et al. 2007.

S.cerevisiae

nr	codons	anticodon	amino acid	D	n	F
1	GCA,GCG	UGC	Ala	0.2571	51437	270.27
2	GCU,GCC	IGC	Ala	0.2571	113163	588.24
3	AGA	UCU	Arg	0.2568	113163	588.24
4	AGG	CCU	Arg	0.2568	10289	54.64
5	CGG	CCG	Arg	0.2568	10289	54.64
6	CGU,CGC,CGA	ICG	Arg	0.2568	61726	322.58
7	AAU,AAC	GUU	Asn	0.2570	102875	555.56
8	GAU,GAC	GUC	Asp	0.2570	154315	833.33
9	UGU,UGC	GCA	Cys	0.2570	41152	217.39
10	CAA	UUG	Gln	0.2569	92589	500.00
11	CAG	CUG	Gln	0.2569	10289	54.64
12	GAA	UUC	Glu	0.2569	144026	769.23
13	GAG	CUC	Glu	0.2569	20574	109.89
14	GGA	UCC	Gly	0.2572	30863	163.93
15	GGG	CCC	Gly	0.2572	20574	109.89
16	GGU,GGC	GCC	Gly	0.2572	164601	909.09
17	CAU,CAC	GUG	His	0.2569	72012	384.62
18	AUA	UAU	Ile	0.2570	20574	109.89
19	AUC,AUU	IAU	Ile	0.2570	133738	714.29
20	CUA,CUG	UAG	Leu	0.2570	30863	163.93
21	CUC,CUU	GAG	Leu	0.2570	10289	54.64
22	UUA	UAA	Leu	0.2570	72012	384.62
23	UUG	CAA	Leu	0.2570	102875	555.56
24	AAA	UUU	Lys	0.2569	72012	384.62
25	AAG	CUU	Lys	0.2569	144026	769.23
26	AUG	CAU	Met	0.2569	51437	270.27
27	UUU,UUC	GAA	Phe	0.2568	102875	555.56
28	CCA,CCG	UGG	Pro	0.2570	102875	555.56
29	CCU,CCC	IGG	Pro	0.2570	20574	109.89
30	AGU,AGC	GCU	Ser	0.2571	41152	217.39
31	UCA	UGA	Ser	0.2571	30863	163.93
32	UCG	CGA	Ser	0.2571	10289	54.64
33	UCU,UCC	IGA	Ser	0.2571	113163	588.24
34	ACA	UGU	Thr	0.2570	41152	217.39
35	ACG	CGU	Thr	0.2570	10289	54.64
36	ACU,ACC	IGU	Thr	0.2570	113163	588.24
37	UGG	CCA	Trp	0.2567	61726	322.58
38	UAU,UAC	GUA	Tyr	0.2568	82300	434.78
39	GUA	UAC	Val	0.2570	20574	109.89
40	GUG	CAC	Val	0.2570	20574	109.89
41	GUU,GUC	IAC	Val	0.2570	144026	769.23

H.sapiens

nr	codons	anticodon	amino acid	D	n	F
1	GCU,GCC,GCA,GCG	IGC,CGC,UGC	Ala	0.2571	1513793	138.89
2	GCU,GCC	IGC	Ala	0.2571	1611009	149.25
3	AGG	CCU	Arg	0.2568	1753677	161.29
4	CGU,CGC	ICG	Arg	0.2568	1326936	121.95
5	AGA	UCU	Arg	0.2568	1762515	161.29
6	CGG,CGA	CCG,UCG	Arg	0.2568	1294110	119.05
7	AAU,AAC	GUU	Asn	0.2570	1128717	104.17
8	GAU,GAC	GUC	Asp	0.2570	1385014	128.21
9	UGU,UGC	GCA	Cys	0.2570	1195632	109.89
10	CAA,CAG	CUG,UUG	Gln	0.2569	1164068	107.53
11	GAA	UUC	Glu	0.2569	1152705	106.38
12	GAA,GAG	CUC,UUC	Glu	0.2569	1060539	98.04
13	GGC,GGU,GGG	CCC,GCC	Gly	0.2572	1204469	111.11
14	GGA	UCC	Gly	0.2572	1482230	136.99
15	CAU,CAC	GUG	His	0.2569	1927909	178.57
16	AUU,AUC	IAU	Ile	0.2570	1353450	125.00
17	AUA	UAU	Ile	0.2570	1871094	172.41
18	UUG	CAA	Leu	0.2570	1492330	136.99
19	CUG	CAG	Leu	0.2570	1623635	149.25
20	UUA,UUG	UAA	Leu	0.2570	1844581	169.49
21	CUU,CUC,CUA	IAG,UAG	Leu	0.2570	1103466	102.04
22	AAG	CUU	Lys	0.2569	1071902	99.01
23	AAA	UUU	Lys	0.2569	1781453	163.93
24	AAA	UUU	Lys	0.2569	1656461	151.52
25	AAA	UUU	Lys	0.2569	1359763	125.00
26	AUG	CAU	Met	0.2569	1592071	147.06
27	UUU,UUC	GAA	Phe	0.2568	1453191	133.33
28	CCU,CCC,CCA,CCG	IGG,CGG,UGG	Pro	0.2570	1160280	107.53
29	UGA	UCA	Sec	0.2570	2011237	185.19
30	UCG	CGA	Ser	0.2571	1881194	172.41
31	AGU,AGC	GCU	Ser	0.2571	1699388	156.25
32	UCU,UCC,UCA	IGA,UGA	Ser	0.2571	1671612	153.85
33	ACG	CGU	Thr	0.2570	1266334	116.28
34	ACU,ACC,ACG	IGU,CGU	Thr	0.2570	1252446	114.94
35	ACA	UGU	Thr	0.2570	1778928	163.93
36	ACA	UGU	Thr	0.2570	1587021	147.06
37	UGG	CCA	Trp	0.2567	2080677	192.31
38	UAU,UAC	GUA	Tyr	0.2568	1367338	126.58
39	GUU,GUC,GUG	IAC,CAC	Val	0.2570	1564295	144.93
40	GUA	UAC	Val	0.2570	1512531	138.89
