# Supplementary material for: Transimulation - Protein Biosynthesis Web Service
Source: PLoS One. 2013 Sep 5;8(9):e73943. doi: 10.1371/journal.pone.0073943 (PMC3764131; doi:10.1371/journal.pone.0073943)
Supplement: Table S3 — The list of codons, their corresponding cognate and near-cognate tRNAs, and competition measures and . (PDF) [file pone.0073943.s005.pdf]

Table S3: The list of codons and their corresponding tRNAs. Column descriptions: (1) codons recognized by a tRNA molecule; (2,3) cognate and near-cognate tRNAs labeled as per Table S2; (4) competition rate C; and (5) competition rate R. Similar table for *E.coli* may be found in the original work of Fluitt et al. 2007.

S.cerevisiae

codon	cognate tRNAs	near-cognate tRNAs	C	R
AAA	24	3,7,18,25,34	5.82	31.51
AAC	7	24,25,30	2.47	23.07
AAG	25	4,7,24,26,35	1.72	16.45
AAU	7	19,24,25,36	4.42	21.12
ACA	34	3,18,24,35,36	7.94	58.89
ACC	36	7,30,34,35	1.78	22.29
ACG	35	4,25,26,34,36	34.77	234.07
ACU	36	19,34,35	1.68	22.39
AGA	3	4,18,24,30,34	1.67	22.39
AGC	30	3,4,7	5.51	61.31
AGG	4	3,25,26,30,35	34.77	234.07
AGU	30	3,4,19,36	8.95	57.88
AUA	18	3,19,24,26,34	19.79	113.39
AUC	19	7,18,26,30	1.61	18.03
AUG	26	4,18,19,25,35	6.30	47.26
AUU	19	18,26,36	1.36	18.29
CAA	10	11,17,20,28	2.32	26.17
CAC	17	10,11,21	1.58	35.75
CAG	11	5,10,17	17.19	251.65
CAU	17	6,10,11,29	2.57	34.77
CCA	28	10,20,29	1.39	24.15
CCC	29	17,21,28	9.05	124.13
CCG	28	5,11,29	0.39	25.15
CCU	29	6,28	7.99	125.19
CGA	6	5,10,20,28	3.95	40.76
CGC	6	5,17,21	1.53	43.18
CGG	5	6,11	6.90	261.93
CGU	6	5,29	0.51	44.20
CUA	20	10,21,28	6.77	82.17
CUC	21	17,20	10.04	258.80
CUG	20	5,11,21	1.00	87.95
CUU	21	6,20,29	10.91	257.92
GAA	12	1,8,13,14,39	1.93	16.24
GAC	8	12,13,16	2.15	14.55
GAG	13	8,12,15,40	16.58	116.60
GAU	8	2,12,13,41	2.68	14.01
GCA	1	2,12,14,39	6.04	47.52
GCC	2	1,8,16	3.42	20.65
GCG	1	2,13,15,40	3.40	50.16
GCU	2	1,41	1.77	22.30
GGA	14	1,12,15,16,39	13.23	75.72
GGC	16	8,14,15	1.22	14.00
GGG	15	13,14,16,40	11.76	121.42
GGU	16	2,14,15,41	1.79	13.43
GUA	39	1,12,14,40,41	18.95	114.23
GUC	41	8,16,39,40	2.55	15.62
GUG	40	13,15,39,41	10.00	123.18
GUU	41	2,39,40	1.05	17.12
UAC	38	9,27	1.78	31.14
UAU	38	33	1.35	31.56
UCA	31	22,32,33	6.27	82.68
UCC	33	9,27,31,32,38	2.42	21.64
UCG	32	23,31,33,37	29.83	239.00
UCU	33	31,32	0.37	23.70
UGC	9	27,37,38	6.04	60.79
UGG	37	9,23,32	2.57	42.14
UGU	9	33,37	4.19	62.64
UUA	22	23,27,31	3.32	34.02
UUC	27	9,22,23,38	2.87	22.67
UUG	23	22,27,32,37	2.37	23.17
UUU	27	22,23,33	2.75	22.79

H.sapiens

codon	cognate tRNAs	near-cognate tRNAs	C	R
AAA	23,24,25	5,7,17,22,35,36	1.93	9.63
AAC	7	22,23,24,25,31	6.68	45.39
AAG	22	3,7,23,24,25,26,33,34	10.95	43.88
AAU	7	22,23,24,25	5.18	46.89
ACA	35,36	5,17,23,24,25,33,34	3.23	13.54
ACC	34	7,31,33,35,36	5.98	41.11
ACG	33,34	3,22,26,35,36	3.11	19.8
ACU	34	33,35,36	3.72	43.37
AGA	5	3,17,23,24,25,31,35,36	7.7	25.58
AGC	31	3,5,7	2.73	31.65
AGG	3	5,22,26,31,33,34	4.93	28.34
AGU	31	3,5	2.06	32.31
AUA	17	5,23,24,25,26,35,36	6.15	24.91
AUC	16	7,17,26,31	4.64	38.58
AUG	26	3,17,22,33,34	4.51	32.07
AUU	16	17,26	2.56	40.67
CAA	10	6,15,21,28	4.72	45.69
CAC	15	10	0.6	29.35
CAG	10	6,15,19,28	5.16	45.25
CAU	15	10	0.6	29.35
CCA	28	6,10,21	3.06	47.35
CCC	28	15	1.66	48.75
CCG	28	6,10,19	3.5	46.91
CCU	28	–	0.0	50.41
CGA	6	10,21,28	2.66	42.77
CGC	4	6,15	2.44	41.89
CGG	6	10,19,28	3.06	42.37
CGU	4	6	0.98	43.35
CUA	21	6,10,19,28	4.74	48.44
CUC	21	15,19	3.21	49.96
CUG	19	6,10,21,28	2.92	33.11
CUU	21	19	1.46	51.71
GAA	11,12	1,8,14,40	2.66	23.38
GAC	8	11,12,13	2.46	39.66
GAG	12	1,8,11,13,39	6.42	48.96
GAU	8	11,12	1.59	40.52
GCA	1	11,12,14,40	3.46	35.34
GCC	1,2	8,13	0.83	17.35
GCG	1	12,13,39	2.55	36.25
GCU	1,2	–	0.0	18.18
GGA	14	1,11,12,13,40	4.33	35.02
GGC	13	8,14	2.39	46.36
GGG	13	1,12,14,39	4.67	44.08
GGU	13	14	1.23	47.52
GUA	40	1,11,12,14,39	4.5	34.3
GUC	39	8,13,40	2.61	34.53
GUG	39	1,12,13,40	3.36	33.78
GUU	39	40	0.96	36.18
UAC	38	9,27	1.92	40.75
UAU	38	–	0.0	42.67
UCA	32	20,29,30	3.43	31.5
UCC	32	9,27,30,38	3.52	31.41
UCG	30	18,32,37	2.8	28.26
UCU	32	30	1.12	33.81
UGA	29	9,20,32,37	3.38	25.47
UGC	9	27,29,37,38	5.8	43.5
UGG	37	9,18,29,30	3.14	24.6
UGU	9	29,37	3.44	45.87
UUA	20	18,27,29,32	3.6	28.02
UUC	27	9,18,20,38	4.07	36.39
UUG	18,20	27,30,37	1.63	15.41
UUU	27	18,20	2.3	38.16
